# Optic Nerve Vascular Compression in a Patient with a Tuberculum Sellae Meningioma

**DOI:** 10.1155/2015/681632

**Published:** 2015-02-01

**Authors:** Cezar José Mizrahi, Samuel Moscovici, Shlomo Dotan, Sergey Spektor

**Affiliations:** ^1^Department of Neurosurgery, Hadassah-Hebrew University Medical Center, 91120 Jerusalem, Israel; ^2^Department of Ophtalmology, Hadassah-Hebrew University Medical Center, 91120 Jerusalem, Israel

## Abstract

*Background*. Optic nerve vascular compression in patients with suprasellar tumor is a known entity but is rarely described in the literature. *Case Description*. We present a unique, well-documented case of optic nerve strangulation by the A1 segment of the anterior cerebral artery in a patient with a tuberculum sellae meningioma. The patient presented with pronounced progressive visual deterioration. Following surgery, there was immediate resolution of her visual deficit. *Conclusion*. Vascular strangulation of the optic nerve should be considered when facing progressive and/or severe visual field deterioration in patients with tumors proximal to the optic apparatus.

## 1. Introduction

It is well known that tuberculum sellae meningiomas cause progressive visual loss by optic nerve compression [1–5], usually due to mechanical compression by the tumor. We present a rare case, well documented, of severe deterioration in visual function as a result of optic nerve strangulation due to compression of the A1 branch of the anterior cerebral artery against the tumor.

## 2. Case Presentation

### 2.1. History and Physical Examination

**Figure 1:**
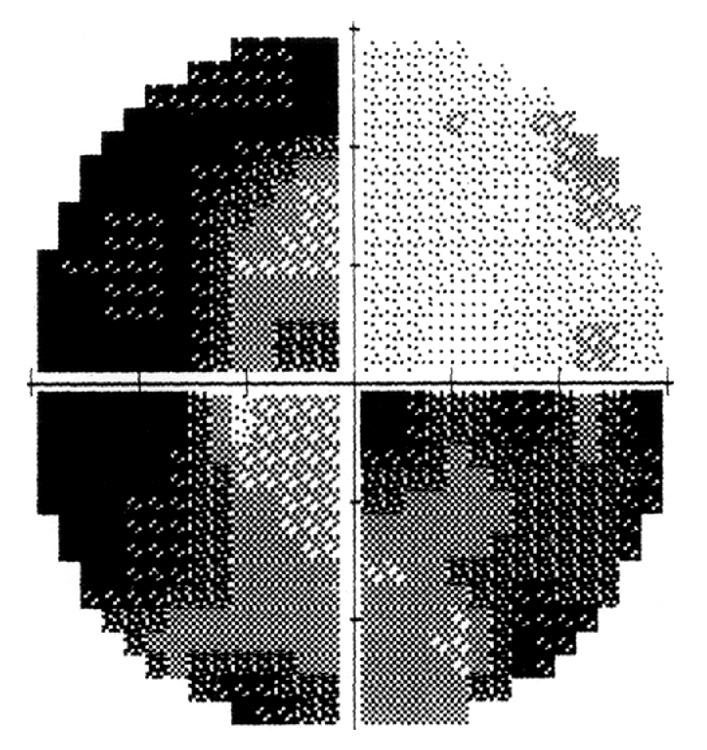
Preoperative left eye visual field. With stimulus V, there was loss of three quadrants with only the superonasal quadrant showing a good degree of preservation.

**Figure 2:**
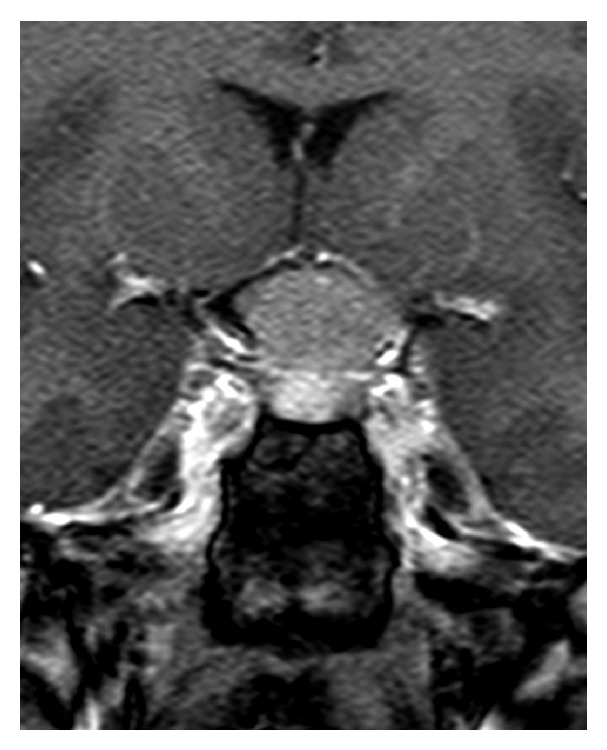
T1-weighted gadolinium-enhanced MRI revealed a tuberculum sellae meningioma measuring approximately 1.7 cm × 1.9 cm × 1.3 cm.

This 50-year-old woman with no known background disease was referred to our Neurosurgery Department for progressive deterioration of visual function in her left eye of 3-4 months duration. Serial visual field examinations with stimulus V showed loss of three quadrants in the left eye, with only the superonasal quadrant showing a good degree of preservation (Figure 1). The right visual field was full, suggesting left optic neuropathy. T1-weighted gadolinium-enhanced MRI revealed a tuberculum sellae meningioma measuring approximately 1.7 cm × 1.9 cm × 1.3 cm (Figure 2).

### 2.2. Surgical Procedure

**Figure 3:**
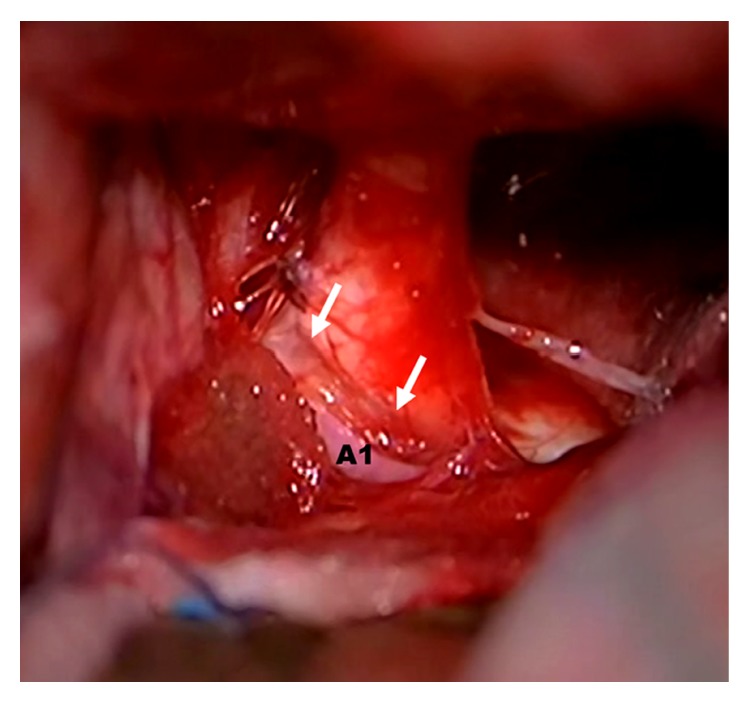
Intraoperative photograph showing a clear impression of the A1 segment after tumor removal and optic nerve vascular decompression.

The patient underwent a left pterional craniotomy. The dura was opened and the Sylvian fissure was split, providing excellent CSF drainage and cerebral relaxation. The left frontal lobe was elevated and the tumor came into view. Inspection of the suprasellar region showed total encasement of the left optic nerve by the tumor. The tumor was internally decompressed, and the anterior communicating complex was released. Tumor pressing upon the left A1 segment of the anterior communicating artery had compressed the left optic nerve. When the tumor was removed, the nerve sagged free, exposing a clear impression of the A1 segment (Figure 3). The capsule and the tumor were then removed completely, and the visual apparatus was decompressed. The patient tolerated the procedure well and was discharged 1 week later.

### 2.3. Followup

**Figure 4:**
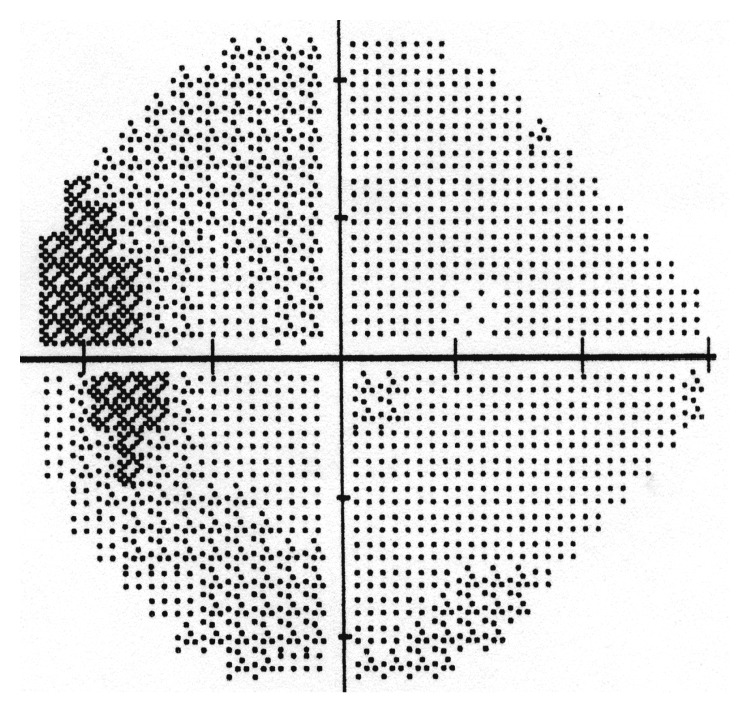
At 1-week postoperative followup, the left eye visual field examination performed with normal stimulus revealed marked improvement but residual central scotoma and temporal depression.

**Figure 5:**
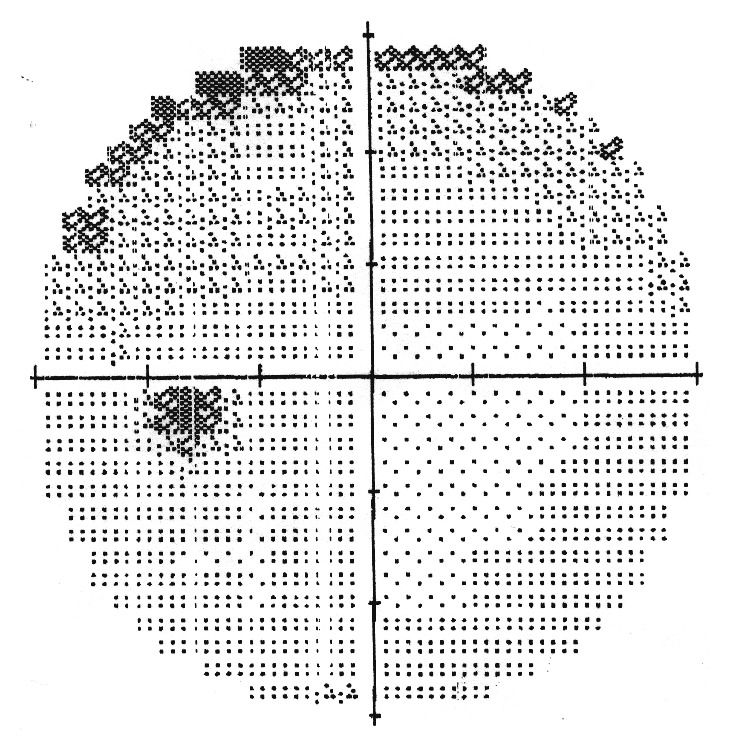
At 10-month postoperative followup, the left eye visual field showed a solitary paracentral scotoma.

**Figure 6:**
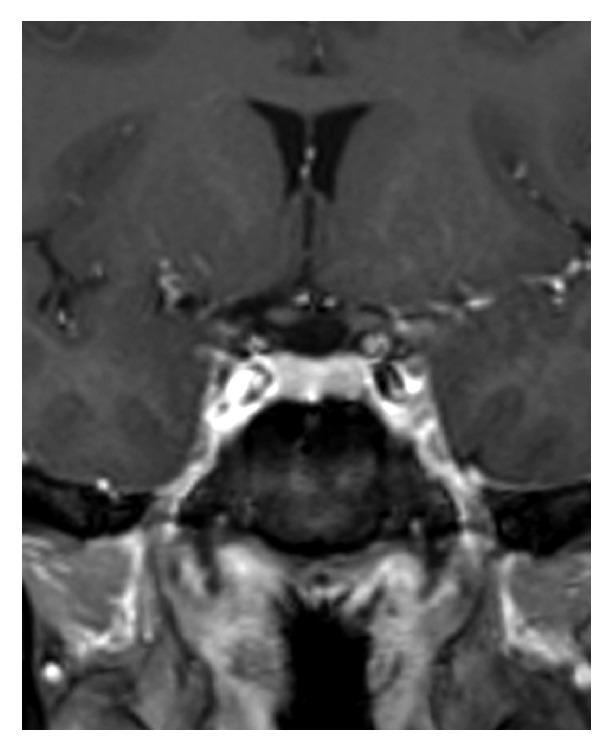
T1-weighted gadolinium-enhanced MRI performed 6 months after surgery showed no evidence of residual tumor.

A full neuroophthalmologic evaluation performed 7 days after surgery revealed best-corrected visual acuity of 0.8 in both eyes with no relative afferent papillary defect. Fundal examination in the right eye showed a pink disc of normal appearance, while in the left eye there was pallor of the temporal part of the optic disc. The right visual field was normal, while in the left visual field there was a central scotoma and temporal depression (Figure 4). Follow-up visual field examination performed 10 months after surgery showed again a normal right visual field and a solitary paracentral scotoma in the left visual field (Figure 5). There was no evidence of residual tumor on MRI performed 6 months after surgery (Figure 6).

## 3. Discussion

We present a well-documented case of optic nerve strangulation by the A1 segment of the anterior cerebral artery in a patient a with suprasellar tumor. Once the tumor was debulked, the optic nerve sagged free presenting a clear impression of the artery, which had compressed the nerve due to pressure exerted by the meningioma on the anterior cerebral artery. Preoperative neuroophthalmic examination revealed a significant deficit in the left visual function. There was dramatic improvement immediately after surgery and near complete resolution in the patient's visual field at 10-month followup.

Visual loss secondary to the mechanical compression of the optic nerve by tumors, particularly by tuberculum sellae meningiomas, is well established in the literature [1–5], and it has been reported that vascular elements may play a significant role in the mechanism of compression [6–8]. Levatin [7] was a pioneer in 1961 when he described strangulation of the optic tract by the anterior cerebral artery in a patient harboring a suprasellar tumor. In 1989, Steno [8] reported compression of structures in the visual pathway by arteries of the circle of Willis within suprasellar tumors in 12 of 34 necropsies of extensive craniopharyngiomas and pituitary adenomas and in three of 109 patients operated on account of these tumors. In addition, there are several reports of compression of the optic nerve by an elongated vascular fusiform enlargement or dolichoectasia [9–12] or a nonaneurysmatic idiopathic artery compression [13, 14].

We found only one paper by Bejjani et al. [6] describing vascular compression of the optic nerve due to pressure exerted by a tuberculum sellae meningioma, with intraoperative illustration of the mechanism of ON strangulation.

Our illustration provides further documentation that this mechanism of strangulation exists and may play role in the pathogenesis of visual loss in patients with infrachiasmatic tumors.

In summary, optic nerve vascular strangulation should be considered when facing progressive and/or severe visual field deterioration patients with tumors proximal to the optic apparatus.
